# Five-year follow-up of the iBerry Study: screening in early adolescence to identify those at risk of psychopathology in emerging adulthood

**DOI:** 10.1007/s00787-024-02462-2

**Published:** 2024-05-22

**Authors:** D. C. Bouter, S. J. Ravensbergen, N. G. M. de Neve-Enthoven, M. Zarchev, C. L. Mulder, W. J. G. Hoogendijk, S. J. Roza, ESPRi Consortium, Wim van Beek, Carla Hagestein-de Bruijn, Mirjam E. J. Kouijzer, Alex J. M. de Ridder, Chi M. van ’t Hooft-Nguyen, Natalie D. Veen, Philip J. S. Michielsen, Mark H. de Jong, N. H. Grootendorst-van Mil

**Affiliations:** 1 Department of Psychiatry, Erasmus MC, University Medical Center Rotterdam, P.O. box 2040, 3000 CA Rotterdam, The Netherlands; 2 Epidemiological and Social Psychiatric Research Institute (ESPRi), Department of Psychiatry, Erasmus MC University Medical Center, Rotterdam, The Netherlands; 3 Parnassia Psychiatric Institute, Rotterdam, The Netherlands

**Keywords:** Epidemiology, Psychopathology, Cohort study, Adolescent, High-risk

## Abstract

The iBerry Study, a Dutch population-based high-risk cohort (*n* = 1022) examines the transition from subclinical symptoms to psychiatric disorders in adolescents. Here, we present the first follow-up measurement, approximately 3 years after baseline assessment and 5 years after the screening based on self-reported emotional and behavioral problems (SDQ-Y). We give an update on the data collection, details on the (non)response, and the results on psychopathology outcomes. The first follow-up (2019–2022) had a response rate of 79% (*n* = 807). Our results at baseline (mean age 15.0 years) have shown the effectiveness of using the SDQ-Y to select a cohort oversampled for the risk of psychopathology. At first follow-up (mean age 18.1 years), the previously administered SDQ-Y remains predictive for selecting adolescents at risk. At follow-up, 47% of the high-risk adolescents showed significant mental health problems based on self- and parent reports and 46% of the high-risk adolescents met the criteria for multiple DSM-5 diagnoses. Compared to low-risk adolescents, high-risk adolescents had a sevenfold higher odds of significant emotional and behavioral problems at follow-up. Comprehensive assessment on psychopathology, substance abuse, psychotic symptoms, suicidality, nonsuicidal self-injury, addiction to social media and/or video gaming, and delinquency, as well as social development, and the utilization of healthcare and social services were conducted. This wave, as well as the ones to follow, track these adolescents into their young adulthood to identify risk factors, elucidate causal mechanisms, and discern pathways leading to both common and severe mental disorders. Results from the iBerry Study will provide leads for preventive interventions.

**Supplementary Information:**

The online version contains supplementary material available at 10.1007/s00787-024-02462-2.

## Introduction

The onset of mental disorders mostly occurs in adolescence and young adulthood. An estimated 62.5% of disorders begin before the age of 25, with a peak age at 14.5 years [1]. These disorders are associated with negative outcomes on educational, occupational, and social domains [2–8]. Compared to those without psychiatric problems, individuals with a psychiatric disorder in their youth are nine times more likely to face negative outcomes on these domains in the transition to adulthood. For youth with subthreshold problems this is five times [9]. Early onset of psychiatric problems is associated with high persistence and negative prognosis [8, 10–12].

The etiology of psychiatric disorders is complex. Studies have shown that psychiatric disorders have a multifactorial etiology and that risk factors are pleiotropic [13–15]. Particularly in adolescence, the symptoms in the early stages of disorders tend to be non-specific [16, 17]. Subsequently, there are high comorbidity rates between psychiatric disorders as well as heterotypic continuity over time [11, 18–20]. This underlines the importance of a transdiagnostic approach in research [21].

To advance a more preventive and transdiagnostic approach in psychiatry, more epidemiological knowledge, especially on individual and environmental exposures, is necessary [7]. This knowledge could be used in individual prediction models for targeted prevention strategies [22, 23]. Considerable effort has been made to study the etiology of psychopathology, but this has been complicated by selective drop-out bias in general population studies, referral bias in patient-based samples, and a focus on a specific diagnosis or inheritance pattern in familial loading studies [24]. More accurate, large-sample deep-phenotype data in a high-risk population is likely to overcome these difficulties [16, 21].

The design of the iBerry (Investigating Behavioral and Emotional Risk in Rotterdam Youth) Study follows a cross-diagnostic approach that cuts across traditional diagnostic boundaries to examine the etiology and course of psychopathology instead of maintaining nosological boundaries with a focus on a single diagnostic category. The main aim of the iBerry Study is to examine the developmental course of psychiatric disorders and associated risk factors to contribute to the development of preventive interventions. The current paper discusses the design and protocol of the first follow-up measurement and gives a cohort profile update, details on the (non) response, and the prevalence of adolescent and parental psychopathology. Furthermore, the long-term effectiveness of using a screening questionnaire to select a cohort oversampled on their self-reported emotional and behavioral problems is discussed.

### Study design

**Fig. 1:**
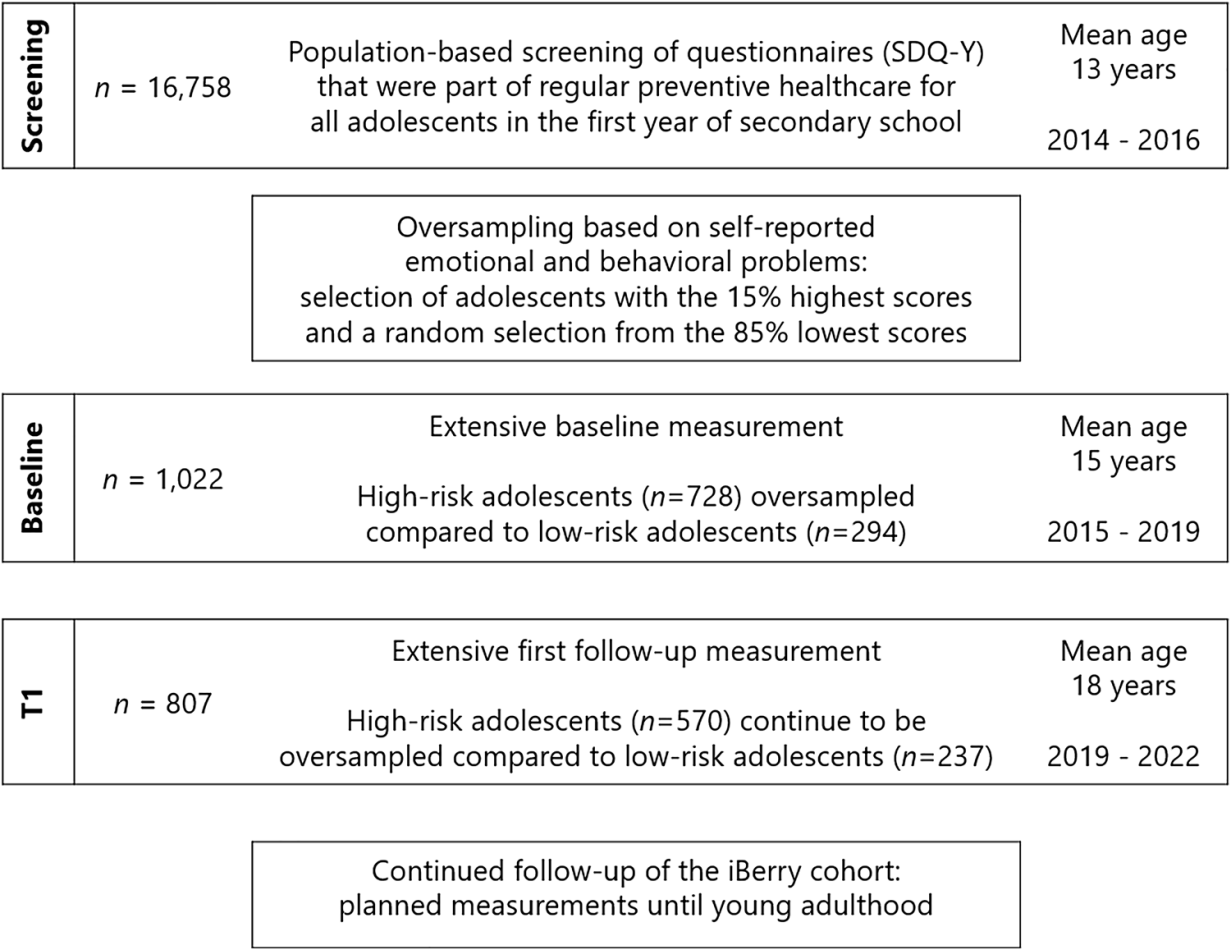
Overview of the design and summary of the different phases of the iBerry Study

The iBerry Study is a cohort study of adolescents from the general population who were oversampled on their self-reported emotional and behavioral problems. The study is conducted in the greater Rotterdam area in the Netherlands, this region contains a combination of the highly urbanized city of Rotterdam, the surrounding suburban cities, and more rural villages [25]. The current study discusses the details from the first follow-up measurement (T1), the screening procedure at age 13 and baseline measurement at age 15 were described in detail elsewhere [24]. A concise graphical overview of the iBerry Study is presented in Fig. 1.

### Eligibility

As described previously by Grootendorst-van Mil and Bouter et al. [24], adolescents were selected for participation in the iBerry Study based on a questionnaire administered in the first year of secondary school as part of standard preventive healthcare performed by community Child and Family Centers in the Netherlands. All adolescents (mean age 13.1 years) filled out the Strengths and Difficulties Questionnaire–Youth (SDQ-Y), to assess their emotional and behavioral problems [26]. Unless the adolescent or their parent(s)/guardian(s) objected, all questionnaires from the school years 2014–2015 and 2015–2016 were screened. From these 16,736 screened questionnaires, adolescents with the highest 15% problem scores were selected, together with a random selection of adolescents with the lower 85% problem scores, resulting in the inclusion of 1,022 adolescents at baseline (September 2015–September 2019, response rate at enrollment 54%).

### Enrollment at the first follow-up

Participants from the baseline measurement were contacted for the first follow-up measurement and 807 adolescents (79.0%) participated at T1. Data were collected between March 2019 and June 2022. Because the COVID-19 pandemic occurred during this measurement, we added two additional online measurements to collect data on emotional and behavioral problems during the lockdowns [27]. The median interval between the SDQ-Y screening and T1 was 4.7 years (IQR 4.5–5.4). The time between baseline and T1 had a median interval of 3.1 years (IQR 3.0–3.5).

### Response rate

215 adolescents (21.0%) included at baseline did not participate at T1. A small number of participants objected to being contacted for follow-up measurements (*n* = 13, 1.3%). 71 adolescents (6.9%) declined participation in the first follow-up. The most common reasons for declining were a lack of interest (*n* = 48, 4.7%) or time (*n* = 10, 1.0%). The remaining 131 adolescents (12.8%) could not be reached.

Response rates were comparable for the high-risk adolescents (78.3%) and low-risk adolescents (80.6%), and the distributions of high-risk and low-risk adolescents were approximately equal in the responders and the non-responders (χ2 = 0.676, *p* = 0.411). A detailed overview of the baseline characteristics of responders and non-responders is provided in Supplementary table S1. Non-responders more often were male, had a higher age at baseline, had a non-Dutch ethnic background, had a lower educational level, and belonged to a lower income household. Non-responders were not more likely to score above the borderline cut-off for emotional and behavioral problems (measured with the Youth Self-Report) at baseline. Significant differences showed small effect sizes. Importantly, those non-responders at T1 were more frequently associated with incomplete baseline measurements, indicating that obtaining comprehensive data from this group was already challenging during the cohort's initial assessment.

### Objectives

The iBerry Study aims to investigate the transition of subclinical symptoms to full-blown psychiatric disorders in adolescents who enter young adulthood. We use a cross-diagnostic approach to study all psychiatric disorders. The aim is to identify risk and protective factors and examine the mechanisms underlying the development of psychopathology.

## Measurements

### Assessment procedure

The adolescents were invited to the research center with one of their parents for a three-hour visit. All participants signed informed consent forms. All researchers were blinded from the SDQ-Y scores during the baseline assessment and follow-up measurements.

Details on data quality, control and management, and privacy protection have been described previously [24]. All protocols have been updated to ensure adherence to all applicable rules and regulations.

### Main outcomes

The main aim is to study the long-term prognosis of adolescent subclinical psychiatric symptoms. We want to examine which determinants can predict the transition of subclinical symptoms to psychiatric disorders. To assess psychopathology we used various methods, including the Achenbach System of Empirically Based Assessment (ASEBA) questionnaires and the MINI Neuropsychiatric interview for DSM-5 diagnoses [28–30]. Specifically, we conducted detailed assessments of psychotic experiences [31], suicidality and nonsuicidal self-injury (NSSI) [32–34], and autism [35]. Subsequent studies will be conducted to examine the various determinants and the in depth measurements of psychopathology.

### Main determinants

A broad range of biological, psychological, and social markers is assessed. At T1 these included socio-demographic characteristics, general functioning, sensory processing, aggressive and delinquent behavior, lifestyle and addiction, health care use and costs, personality, coping, family functioning, parenting, peers, relationships, sexuality, (adverse) life events and trauma, neuropsychological functioning, somatic complaints, anthropometry, and puberty development. We also collected detailed information on sleep and movement which was assessed using nine-day actigraphy measurements and a daily diary. Parents also provided information on parental psychopathology, personality, substance use, and anthropometry. A complete overview of all measurements is provided in Supplementary table S2.

### Biological samples

Biological measurements at baseline were repeated at T1; we again obtained a blood and a hair sample from both the adolescent and from the accompanying parent.

## Characteristics of the study cohort

### Socio-demographic characteristics

Socio-demographic characteristics of the adolescents and their parents are presented in Table 1. At the first follow-up the adolescents in the cohort had a mean age of 18.1 years, 53.5% were female, 23.6% had a non-Dutch ethnic background, 61% lived in an urban area, and vocational education was the most reported education level. For 85.1% of the adolescents, one of their parents (83.4% mothers) also participated in the study. High-risk and low-risk adolescents differed on educational level, whether they participated with a parent, parental education level, and household income level, albeit with small effect sizes. The oversampled ratio of high-risk to low-risk adolescents remained consistent between baseline (2.5:1) and T1 (2.4:1), 570 high-risk adolescents (70.6%) and 237 low-risk adolescents (29.4%) participated at T1.

**Table 1:** Socio-demographic characteristics of the participating adolescents and parents at first follow-up

	Total cohort (*n* = 807)		High-risk (*n* = 570)		Low-risk (*n* = 237)		
	*n*	%	*n*	%	*n*	%	
Adolescents							
Sex, female (%)	432	53.5	303	53.2	129	54.4	*p* = .757, φ = − 0.012
Age (*M*, *SD*)	18.1	0.86	18.1	0.87	18.1	0.84	*p* = .751,* d* = 0.025
Ethnic background							*p* = .193, V = 0.130
Dutch	614	76.4	436	76.9	178	75.1	
Other Western	49	6.1	33	5.8	16	6.8	
Asian	34	4.2	21	3.7	13	5.5	
African	11	1.4	7	1.2	4	1.7	
South-American	11	1.4	11	1.9	–	–	
Surinamese	38	4.7	23	4.1	15	6.3	
Moroccan	8	1.0	7	1.2	1	0.4	
Turkish	8	1.0	6	1.1	2	0.8	
Dutch Antilles	18	2.2	11	1.9	7	3.0	
Cape Verdean	13	1.6	12	2.1	1	0.4	
Urbanicity							*p* = .481, V = 0.042
Rural	174	21.5	118	20.7	56	23.6	
Suburban	141	17.5	97	17.0	44	18.6	
Urban	492	61.0	355	62.3	137	57.8	
Education level							*p* < .001, V = 0.242
Secondary education, low	60	7.6	51	9.2	9	3.9	
Secondary education, medium	106	13.5	67	12.0	39	16.9	
Secondary education, high	134	17.0	74	13.3	60	26	
Higher education, low	357	45.4	285	51.3	72	31.1	
Higher education, medium	105	13.3	66	11.9	39	16.9	
Higher education, high	25	3.2	13	2.3	12	5.2	
Parents							
Adolescent participating with parent at T1	687	85.1	476	83.5	211	89.0	*p* = .045, φ = − 0.071
Sex of the parent, female	573	83.4	395	83.0	178	84.4	*p* = .683, φ = − 0.016
Age of the parent (*M*, *SD*)	49.8	5.48	49.7	5.60	50.2	5.17	*p* = .231,* d* = 0.104
Ethnic background of the parent							*p* = .397, V = 0.127
Dutch	534	81.5	379	82.8	155	78.7	
Other Western	45	6.9	26	5.7	19	9.6	
Asian	21	3.2	12	2.6	9	4.6	
African	3	0.5	2	0.4	1	0.5	
South-American	8	1.2	6	1.3	2	1.0	
Surinamese	21	3.2	14	3.1	7	3.6	
Moroccan	2	0.3	2	0.4	–	–	
Turkish	5	0.8	4	0.9	1	0.5	
Dutch Antilles	8	1.2	5	1.1	3	1.5	
Cape Verdean	8	1.2	8	1.7	–	–	
Education level of the parent							*p* = .004, V = 0.141
Low (primary education)	8	1.2	6	1.3	2	1.0	
Intermediate (secondary school, vocational training)	388	57.2	291	61.4	97	47.5	
High (bachelor's degree)	180	26.6	118	24.9	62	30.4	
University	102	15.0	59	12.4	43	21.1	
Net monthly household income							*p* < .001, V = 0.169
≤ € 1599	34	5.3	22	4.8	12	6.1	
€ 1600–2399	82	12.6	68	15.0	14	7.1	
€ 2400–4399	283	43.6	210	46.4	73	37.3	
≥ € 4400	250	38.5	153	33.8	97	49.5	

### Emotional and behavioral problems

Emotional and behavioral problems, as measured with the Youth Self-Report (YSR) and Child Behavior Checklist (CBCL), are described in Table 2. In the high-risk group 37.1% of the adolescents reported problems in the borderline/clinical range (ASEBA norm scores, > 93rd percentile [28]), compared to 16.0% of the adolescents in the low-risk group. Combining the multi-informant questionnaires, 40.0% of the adolescents in the cohort (47.0% in the high-risk group, 23.1% in the low-risk group) scored above the borderline cut-off on the Total Problems scale according to one or both informants. For all scales, more high-risk adolescents scored above the cut-off compared to the low-risk adolescents.

**Table 2:** Emotional and behavioral problems of the adolescents participating at T1

	Total (n = 802)		High-risk (n = 562)		Low-risk (n = 231)		
	Median (range)	Percentage above borderline cut-off	Median (range)	Percentage above borderline cut-off	Median (range)	Percentage above borderline cut-off	
Self-report by the adolescent^a^							
Internalizing problems	12 (0–50)	36.1	14 (0–51)	41.8	9 (0–36)	22.1	*p* < .001, φ = − 0.187
Externalizing problems	9 (0–39)	16.7	10 (0–39)	19.8	7 (0–31)	9.1	*p* < .001, φ = − 0.130
Total problems	41 (1–142)	30.9	46 (3–142)	37.1	32 (1–86)	16.0	*p* < .001, φ = − 0.207
Reported by the parent who accompanied the adolescent^b^							
Internalizing problems	6 (0–51)	29.3	8 (0–51)	34.0	4 (0–44)	18.7	*p* < .001, φ = − 0.155
Externalizing problems	4 (0–61)	13.1	5 (0–61)	15.7	2 (0–27)	7.2	*p* < .001, φ = − 0.117
Total problems	19 (0–191)	23.5	24 (0–191)	28.1	12 (0–73)	12.9	*p* < .001, φ = − 0.165
Multi-informant, % one or both above borderline cut-off							
Internalizing problems		45.9		52.3		30.3	*p* < .001, φ = − 0.200
Externalizing problems		22.4		26.1		13.7	*p* < .001, φ = − 0.135
Total problems		40.0		47.0		23.1	*p* < .001, φ = − 0.222

^a^Measured with the Youth-Self Report, which was missing for 14 adolescents

^b^Measured with the Child Behavior Checklist, which was missing for 121 adolescents

### Adolescent psychopathology

In 729 adolescents a complete semi-structured interview for the DSM-5 diagnoses was conducted (Table 3). The most common diagnoses in the high-risk group were substance-related disorders (40.0%), followed by mood disorders (35.1%), and ADHD (29.5%). In the low-risk group, substance-related disorders were common (42.1%), followed by mood disorders (16.8%). Notably, the high-risk adolescents more often met the criteria for multiple diagnoses (46.0%) compared to the low-risk adolescents (24.8%).

**Table 3:** Current psychopathology (last 2 years) in adolescents and one of their parents, assessed using structured clinical DSM interviews at T1

	Adolescent psychopathology						Parental psychopathology					
	Total n = 729		High-risk n = 515		Low-risk n = 214		Total n = 545		High-risk n = 375		Low-risk n = 170	
Mood disorders	217	29.8	181	35.1	36	16.8	72	13.2	57	15.2	15	8.8
Anxiety disorders	181	24.8	151	29.3	30	14.0	70	12.9	52	13.9	18	10.6
Obsessive–compulsive disorders	40	5.5	35	6.8	5	2.3	14	2.6	11	3.0	3	1.8
Posttraumatic stress disorders	27	3.7	26	5.0	1	0.5	4	0.7	4	1.1	0	0.0
Substance use disorders	296	40.6	206	40.0	90	42.1	23	4.2	18	4.8	5	2.9
Psychotic disorders	40	5.5	35	6.8	5	2.3	18	3.3	12	3.2	6	3.5
Attention deficit hyperactivity disorder	174	23.9	152	29.5	22	10.3	0	0.0	0	0.0	0	0.0
Disruptive behavior disorders	58	8.0	48	9.3	10	4.7	3	0.6	2	0.5	1	0.6
Tic disorders	21	2.9	17	3.3	4	1.9	–	–	–	–	–	–^a^
Eating disorders	21	2.9	19	3.7	2	0.9	2	0.4	2	0.5	0	0
Somatoform disorders	–	–	–	–	–	–^a^	27	5.0	22	5.9	5	2.9
Premenstrual dysphoric disorder	–	–	–	–	–	–^a^	24	4.4	16	4.3	8	4.7
Adjustment disorders	27	3.7	18	3.5	9	4.2	51	9.4	36	9.7	15	8.8
No psychopathology	216	29.6	133	25.8	83	38.8	334	61.3	219	58.4	115	67.6
One diagnosis	223	30.6	145	28.2	78	36.4	145	26.6	104	27.7	41	24.1
Multiple diagnoses	290	39.8	237	46.0	53	24.8	66	12.1	52	13.9	14	8.2

^a^Diagnoses were not part of the diagnostic interview

### Parental psychopathology

In line with the prevalences presented in our design paper [24], parents in the cohort showed higher rates of psychopathology in the past 2 years compared to the general population (Table 3). In the parents of high-risk adolescents 27.7% met the criteria for one DSM-IV diagnosis, 13.9% met the criteria for multiple DSM-IV diagnoses. In the parents of low-risk adolescents, these prevalences were 24.1% and 8.2%, respectively. Because a validated Dutch translation of the DSM-5 interview was not available at the start of T1, the same DSM-IV interview from the baseline measurement was used for consistency.

## Predictiveness of SDQ-Y score

**Fig. 2:**
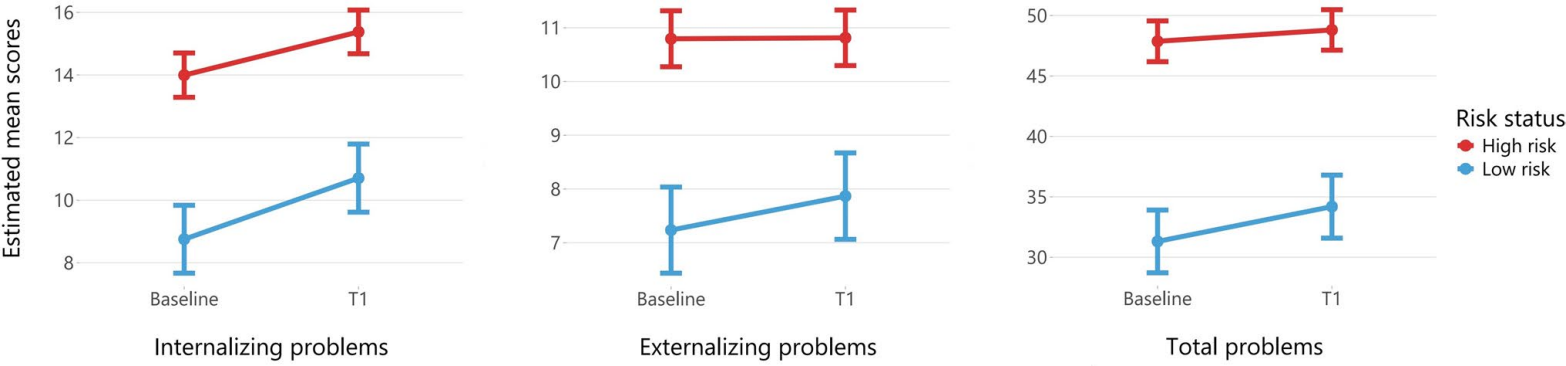
Estimated emotional and behavioral problem mean scores for the internalizing and externalizing subscales and the total problem scale at baseline and first follow-up measurement (T1) stratified by risk status

To investigate the longitudinal predictive value of the SDQ-Y score at age 13 on the development of emotional and behavioral problems, we used multilevel random intercept regression models. We used sex, age, time, and an interaction term between time and risk status to model self-reported internalizing, externalizing, and YSR Total Problems scores. The estimated mean scores from linear mixed models for the three problem scales, stratified by SDQ-Y risk status, are visualized in Fig. 2. The coefficient estimates are summarized in Supplementary table S3. Next, we examined adolescents who reported internalizing, externalizing, and total problems above the borderline cut-off score. The odds ratios from the mixed effect logistic regression are presented in Table 4. Both in the linear and logistic models, we did not find an interaction effect between time and risk status. Between baseline and T1, the trajectory of emotional and behavioral problems did not differ between low and high-risk adolescents. Overall, both groups either remained equal or increased on emotional and behavioral problems. Notably, the difference between low and high-risk adolescents remained stable between baseline and T1, indicating that the SDQ-Y score is a good predictor of later psychopathology both in the short-term (baseline, after ~ 1–3 years) and in the medium-long term (T1, after ~ 4–6 years). Adolescents identified as high-risk had a four to sevenfold higher odds of scoring in the borderline range on internalizing, externalizing, and total problems compared to low-risk adolescents.

**Table 4:** Odds ratios from mixed effect logistic regression models including a random intercept, time, risk status, age, and sex to model adolescents reporting internalizing, externalizing, and total problems in the borderline/clinical range

	Internalizing problems			Externalizing problems			Total problems		
	Odds ratio	95% CI	p	Odds ratio	95% CI	p	Odds Ratio	95% CI	p
(Intercept)	0.01	0.00, 0.14	< .001	< 0.00	0.00, 0.07	.007	0.02	0.00, 0.40	.009
Time [T1]	**2.17**	**1.26, 3.72**	**.005**	1.53	0.95, 2.47	0.082	**2.28**	**1.22, 4.26**	**.010**
Risk status [high]	**4.50**	**2.68, 7.57**	< **.001**	**4.78**	**1.64, 13.94**	**.004**	**7.22**	**3.93, 13.28**	< **.001**
Age	1.13	0.95, 1.34	.160	1.09	0.70, 1.69	.711	1.03	0.86, 1.23	.755
Sex [female]	**1.87**	**1.37, 2.57**	< **.001**	1.53	0.67, 3.49	.316	**1.49**	**1.07, 2.09**	**.020**
Time [T1] × risk status [high]	0.76	0.41, 1.40	.383	–^a^	–^a^	–^a^	0.62	0.31, 1.24	.179

^a^Due to low numbers of low-risk adolescents in the borderline/clinical range at T1 the model did not converge, a simplified model without the interaction term is presented

Significant results are presented in bold

## Statistical power

In future studies that make use of the collected data, not all analyses will be performed in the complete cohort because of loss to follow-up and missing values. Depending on the association under study, we will consider the information that is available for the main predictor and main outcome variable. Based on sample sizes ranging from 1000 to 500 (with an alpha value of 0.05 and 80% power), the study can detect a difference in standard deviation ranging from 0.18–0.25 (50% prevalence) to 0.41–0.58 (5% prevalence). These are detectable effect sizes using a dichotomous measure of exposure, which are considered conservative. Within the iBerry Study, we will often study the effect of continuous determinants and prognostic factors, assessed at multiple time points, which will further increase power.

## Follow-up and retention strategies

All adolescents received gift certificates for their participation. All travel expenses were reimbursed. To keep in contact we send newsletters, birthday cards, and holiday cards. It is also possible to follow the study on social media channels. To minimize loss to follow-up we ensured the correctness of contact information at each contact. If an adolescent was unable to participate it was possible to make an appointment during the evening, online, as a home visit, or to postpone the visit for a couple of months. Lastly, if these possibilities were not an option a short questionnaire was sent to the adolescent to collect data on sociodemographic characteristics, emotional and behavioral problems, psychotic experiences, (adverse) life events, substance use, and self-harm. In a separate questionnaire parents provided additional information on emotional and behavioral problems, adverse life events, healthcare use, personality, executive functioning, and family functioning.

## Data linkage and collaboration

The available information of the participants makes it possible to integrate the cohort data with other data sources. Environmental characteristics can be studied based on the home address to be linked with data from Statistics Netherlands or in collaboration with the Geoscience and health consortium [27, 36]. Furthermore, adolescents provided informed consent to obtain additional information on their health and development from their healthcare providers. We warmly welcome other researchers to collaborate by combining the iBerry cohort data with other studies.

## Strengths and limitations

Our results show that the screening procedure was successful in selecting a cohort of adolescents at risk of psychopathology. Adolescents with a high score on emotional and behavioral problems at age 13 were four to seven times more likely to report significant emotional and behavioral problems at age 18. New normative SDQ-Y norms for Dutch adolescents showed that the 15% SDQ-Y cut-off used to select the high-risk population aligns with adolescents scoring above the borderline/clinical cut-off score [37]. This relative cut-off score where adolescents were compared to their peers, is an efficient way to select adolescents at risk for psychopathology as evidenced by the high levels of psychopathology in our cohort at follow-up.

The specific design of our cohort enables us to address a limitation of general population studies that suffer from drop-out dependent on less prevalent risk factors and outcomes. With the oversampled selection and the retention of adolescents at higher risk of psychopathology, we will continue to have the advantage of increased power to examine associations of interest. Despite oversampling individuals with a high risk for psychopathology, the risk factors that our study identifies are relevant to the general population as well. This enriched population might enable us to pick up risk factors that are more rare, but it is not likely that the oversampling will lead to spurious associations [38].

Although our retention rate is high, we still have adolescents who were lost to follow-up. As indicated by our non-response analyses, these adolescents do differ on socio-demographic characteristics from the adolescents who did participate. This could introduce bias in our results, which we will reduce as much as possible. Attrition will be addressed in a targeted manner for each association under study, for example by controlling analyses for potential confounders and by using multiple imputation methods [39–44]. Furthermore, the scientific inference of our findings will not necessarily be compromised. While a completely representative sample of the source population is valuable, broader scientific generalizations can sometimes hold more significance than strict sample representativeness, especially when examining associations between variables of interest [45]. Selective drop-out will likely result in biased estimations of prevalences, and by design the prevalences within the iBerry cohort will be higher than in the general population, but this will not likely affect the direction of the associations under study [46–48].

## Future perspectives

Now that the cohort has been established and we have found successful retention strategies, we aim to follow up this cohort until young adulthood. Adolescents are invited for follow-up visits every 2–3 years. Currently, the third visit to the research center is being conducted. This visit includes both repeated as well as new age-appropriate measurements, such as an extensive assessment for personality disorders.

To ensure valid conclusions we will also request microdata, anonymized data at the level of private individuals from Statistics Netherlands (CBS), which enables us to study the outcome in both individuals participating in subsequent follow-up phases and in those who might be lost to follow-up.

## Conclusion

The iBerry Study closely examines adolescents and their parents to determine a broad range of biological, psychological, and social markers for the transition of subclinical symptoms to psychiatric disorders. Adolescents were successfully selected based on their self-reported emotional and behavioral problems at age 13. The current data underscore an evident vulnerability in high-risk adolescents, manifesting in significant psychopathological problems by age 18. The research's in-depth measurements in combination with its design, anchored by its careful screening and retention strategies, create a foundation for numerous detailed future investigations into the complexities of adolescent psychopathology.

## Supplementary Information

Below is the link to the electronic supplementary material.

Supplementary file1 (PDF 421 KB)

## Data Availability

Other researchers are welcome to collaborate with researchers in the iBerry Study group and to request access to the data. Proposals to collaborate will be assessed by the iBerry Study group with respect to quality, feasibility, and potential overlap with planned or published publications.
