# Supplementary material for: Five-year follow-up of the iBerry Study: screening in early adolescence to identify those at risk of psychopathology in emerging adulthood
Source: Eur Child Adolesc Psychiatry. 2024 May 22;33(12):4285–94. doi: 10.1007/s00787-024-02462-2 (PMC11618212; doi:10.1007/s00787-024-02462-2)
Supplement: Supplementary file 1 — Supplementary file1 (PDF 421 KB) [file 787_2024_2462_MOESM1_ESM.pdf]

Supplementary tables

Supplementary table S1. Characteristics at baseline of the adolescents who participated in the T1 measurement and the adolescent who did not participate at T1

Baseline characteristics	T1 Responders (n=807, 79.0%) n	%	T1 Non-responders (n=215, 21.0%) n	%	
Sex, female	432	53.5	90	41.9	*p*=.002, φ=-.095
Age (*M, SD*)	14.9	0.91	15.3	0.97	*p*<.001, *d*= .389
SDQ-Y status, high risk	570	70.7	158	73.1	*p*=.411, φ=-.026
Ethnic background					*p*<.001, V=.237
Dutch	589	79.3	120	69.8	
Asian	25	3.4	5	2.9	
African	6	0.8	1	0.6	
South-American	6	0.8	-	-	
Other Western	47	6.3	8	4.7	
Surinames	34	4.6	15	8.7	
Moroccan	5	0.7	7	4.1	
Turkish	5	0.7	7	4.1	
Dutch Antilles	14	1.9	5	2.9	
Cape Verdean	12	1.6	4	2.3	
Missing at baseline	63	7.8	43	20.0	
Education level					*p*<.001, V=.262
Special needs secondary education	21	2.7	15	8.1	
Pre-vocational secondary education	325	42.0	106	57.0	
Higher general secondary education	186	24.0	33	17.7	
Pre-university education	176	22.7	10	5.4	
Combined education level	66	8.5	22	11.8	
Missing	33	4.1	29	13.5	
Net monthly household income					*p*<.001, V=.222
≤ € 1599	68	9.6	40	24.5	
€ 1600-2399	106	14.9	32	19.6	
€ 2400-4399	371	52.3	63	38.7	
≥ € 4400	165	23.2	28	17.2	
Missing at baseline	97	12.0	52	24.2	
Urbanicity					*p*=.298, V=.049
Rural	173	21.4	36	16.7	
Suburban	148	18.3	44	20.5	
Urban	486	60.2	135	62.8	
YSR internalizing problems score *(Median, IQR)*	11	6-17	9	5-9	*p*<.006, *r*= .089
YSR externalizing problems score (*Median, IQR*)	9	5-13	9	5-9	*p*<.281, *r*= -.035
YSR total problems score *(Median, IQR)*	40	28-54	36	24-36	*p*<.040, *r*= .066
Internalizing, % above borderline cut-off	209	26.9	46	24.5	*p*=.492, φ=.022
Externalizing, % above borderline cut-off	113	14.5	30	16.0	*p*=.605, φ=-.017
Total problems, % above borderline cut-off	191	24.5	42	22.3	*p*=.542, φ=.020
YSR missing at baseline	26	3.2	27	12.6	*p*<.001, φ=.172

Supplementary table S2. Overview of all assessments

Assessment of the adolescent	**Instrument**	**Abbreviation**	**Type**	**Baseline**	**T1**
General characteristics	Demographic characteristics		Questionnaire	A	A, P
	Pregnancy, development during childhood, current health		Questionnaire	P	
	Financial situation, including Psychological Inventory of Financial Scarcity [1]	PIFS	Questionnaire		A
	Religion		Questionnaire		A
	Ethnicity, self-identification		Questionnaire		A
	Educational attainment		Questionnaire	A	A
General functioning	Child Outcome Rating Scale [2, 3]	CORS	Self-report	A, P, R	A, P, R
	Children's Global Assessment Scale [4]	CGAF	Assessment	R	R
	Brief Psychiatric Rating Scale for Children [5, 6]	BPRS-C	Assessment	R	R
	Pediatric Quality of Life Scale [7]	PedsQL	Questionnaire	A	
Psychopathology	Youth Self-Report [8, 9]	YSR	Questionnaire	A	A
	Child Behavior Checklist 6-18 [8, 9]	CBCL	Questionnaire	P*	P
	Teacher's Report Form [8, 9]	TRF	Questionnaire	T	
Psychiatric disorders	Mini Neuropsychiatric Interview for Children and Adolescents [10, 11]	MINI-KID	Interview	A	A
Psychotic symptoms	Prodromal Questionnaire 16 [12]	PQ-16	Questionnaire	A	A
Suicidality and self-harm	Inventory of Statements about Self-Injury [13]	ISAS	Questionnaire	A	A
	Questionnaire about suicidality and self-injury [14]	VOZZ-SCREEN	Questionnaire	A	A
	Columbia-Suicide Severity Rating Scale [15]	C-SSRS	Interview		A
Autism spectrum disorders	Social Responsiveness Scale [16, 17]	SRS-2	Questionnaire		P
Sensory processing	Adolescent/Adult Sensory Profile [18, 19]	AASP	Questionnaire		A
Aggressive and delinquent behavior	Self-Reported Early Delinquency Scale [20]	SRED	Interview	A	A
	Reactive-Proactive Aggression Questionnaire [21, 22]	RPQ	Questionnaire		A
Lifestyle and addiction	Substance use		Questionnaire	A	A
	Drinking Motive Questionnaire – Revised – Short Form [23]	DMQ-R-SF	Questionnaire		A
	Compulsive Internet Use Scale [24]	CIUS	Questionnaire		A
	Social media stress [25]	SMS	Questionnaire	A	A
	Videogame addiction test [26]	VAT	Questionnaire	A	A
Psychopathy	Youth Psychopathic traits Inventory- Short Child Version [27]	YPI-SCV	Questionnaire	A	
Temperament	Early Adolescent Temperament Questionnaire – Revised [28, 29]	EATQ-R	Questionnaire	A	

Health care use and costs	Trimbos/iMTA questionnaire on Costs associated with Psychiatric illness [30]	TiC-P	Interview	P	P
Self	Sense of Coherence [31, 32]	SOC-13	Questionnaire	A	
	Rosenberg Self-Esteem Scale [33]	RSES	Questionnaire	A	
	COPE inventory [34]	COPE-easy	Questionnaire		A
Personality	McLean Screening Instrument for Borderline Personality Disorder [35, 36]	MSI-BPD	Questionnaire		A
	Personality Inventory for DSM-5 [37, 38]	PID-5 SF	Questionnaire		P
Family functioning	Family Assessment Device, General Functioning [39, 40]	GF12/GF6+	Questionnaire	P	P
	Parenting burden questionnaire [41]	OBVL	Questionnaire		P
Parenting	Conflict Tactics Scale Parent-Child [42]	CTSPC	Questionnaire	A, P	
	Parent-child interaction questionnaire [43]	OKIV	Questionnaire	A, P	P
	Questionnaire family functioning for parents [44]	VGFO	Questionnaire		P
	Parental supervision/involvement scale [45]	S/IS-R	Questionnaire		P
Peers	Bullying questionnaire		Questionnaire	A	A
Relationships	Multidimensional Scale of Perceived Social Support [46]	MSPSS	Questionnaire	A	A
Life events	Major life events interview (P) / questionnaire (A)		Interview	P	P, A
Trauma	Child Trauma Questionnaire - Short Form [47, 48]	CTQ-SF	Questionnaire		A
Sexuality	Sexual experiences		Questionnaire		A
	Sexual orientation		Questionnaire		A
	Risky sexual behavior		Questionnaire		A
	Abortion / pregnancy		Questionnaire		A
Neuropsychological functioning	IQ - Snijders-Oomen Non-verbal Intelligence test [49]	SON-R	Test	A	
Risky decision making	IOWA Gambling task [50, 51]	IOWA GT	Test	A	
Executive functioning	Behavior Rating Inventory for Executive Function – Screener [52]	BRIEF-screen	Questionnaire		P
Somatic complaints	Physical Complaints Questionnaire [53]	LKV	Interview	A	
Audio sample	Five Minute Speech Sample [54]	FMSS	Interview	A, P	A, P
Anthropometry	Waist circumference		Assessment	A	A
Height and weight	Body Mass Index	BMI	Assessment	A	A
Body image			Questionnaire	A	
Puberty development	Questionnaire and Tanner stadia		Questionnaire	A	A
Biological samples	Blood sample		Sample	A	A
	Hair sample		Sample	A	A
Advanced glycation end-products	Advanced glycation end-products reader (Skin-autofluorescence) [55]	AGE-reader	Assessment		A

Thermal sensory testing	Quantitative sensory testing [56]	QST	Assessment		A
Sleep and movement	Actigraphy		Actiwatch		A
	Sleep diary		Questionnaire		A
	Sleep and movement questionnaire		Questionnaire		A

Assessment of the parent	**Instrument**	**Abbreviation**	**Type**	**Baseline**	**T1**
General characteristics	Demographic characteristics		Questionnaire	P*	P
	Health		Questionnaire	P*	
	Family situation		Questionnaire		P
	Financial situation, including Psychological Inventory of Financial Scarcity [1]	PIFS	Questionnaire	P	P
	Religion		Questionnaire		P
	Ethnicity, self-identification		Questionnaire		P
General functioning	Global Assessment of Functioning [57]	GAF	Assessment	R	R
Psychopathology	Mini Neuropsychiatric Interview [58, 59]	MINI-PLUS	Interview	P	P
	Brief Symptom Inventory [60]	BSI	Questionnaire	P*	P
	Prodromal questionnaire 16 [12]	PQ-16	Questionnaire		P
	Inventory of Statements about Self-Injury [13]	ISAS	Questionnaire		P
	Questionnaire about suicidality and self-injury [14]	VOZZ-screen			
Personality	Standard Assessment of Personality Abbreviated Scale [61, 62]	SAPAS	Questionnaire	P*	
	McLean Screening Instrument for Borderline Personality Disorder [35, 36]	MSI-BPD	Questionnaire		P
	Personality Inventory for DSM-5 [37]	PID-5 SF	Questionnaire		P
Substance use			Questionnaire	P*	P
Neuropsychological functioning	Snijders-Oomen Non-verbal Intelligence test [49]	SON-R	Test	P	
Health care use and costs	Treatment inventory Cost in Psychiatric patients [63]	TiC-P	Interview	P	
Audio sample	Five Minute Speech Sample [54]	FMSS	Interview	P	P
Anthropometry	Waist circumference		Assessment	P	P
Height and weight	Body Mass Index	BMI	Assessment	P	P
Biological samples	Blood sample		Sample	P	P
	Hair sample		Sample	P	P

A = Adolescent, P = Parent, R = Researcher, T = Teacher

* Where possible, these measurements were also assessed from the second parent or caregiver at baseline

Supplementary table S3. Summary of the coefficient estimates from mixed effect linear regression models for internalizing, externalizing, and total problems reported by the adolescents

	**Internalizing problems**			**Externalizing problems**			**Total problems**		
	Coefficient	*95% CI*	*p*	*Coefficient*	*95% CI*	*p*	*Coefficient*	*95% CI*	*p*
(Intercept)	-0.31	-8.76, 8.13	.942	4.99	-1.35, 11.34	.123	23.44	3.18, 43.71	.023
Time [T1]	**1.95**	**0.82, 3.09**	**< .001**	0.63	-0.16, 1.43	.119	**2.88**	**0.19, 5.57**	**.036**
Risk status [High]	**5.24**	**3.95, 6.53**	**< .001**	**3.56**	**2.60, 4.51**	**< .001**	**16.56**	**13.46, 19.65**	**< .001**
Age	0.39	-0.16, 0.95	.164	0.15	-0.27, 0.57	.478	0.27	-1.06, 1.61	.687
Sex [Female]	**6.35**	**5.34, 7.36**	**< .001**	-0.03	-0.79, 0.73	.939	**7.55**	**5.13, 9.97**	**< .001**
Time [T1] × Risk status [High risk]	-0.57	-1.92, 0.78	.406	-0.61	-1.56, 0.33	.204	-1.94	-5.15, 1.27	.236

References

1. van Dijk, W.W., M.M.B. van der Werf, and L.F. van Dillen, *The Psychological Inventory of Financial Scarcity (PIFS): A psychometric evaluation.* Journal of Behavioral and Experimental Economics, 2022. 101: p. 101939.
2. Boon, A., S. De Boer, and E. Ravensteijn, *De Child outcome rating scale (C-ORS) en de child session rating scale (C-SRS): Het belang van de therapeutische alliantie voor het behandelresultaat.* Tijdschrift voor psychotherapie, 2012. 38: p. 73-87.
3. Duncan, B.L., et al., *Giving Youth a Voice: A Preliminary Study of the Reliability and Validity of a Brief Outcome Measure for Children, Adolescents, and Caretakers.* Journal of brief therapy, 2006. 5(2): p. 66-82.
4. Shaffer, D., et al., *A children's global assessment scale (CGAS).* Arch Gen Psychiatry, 1983. 40(11): p. 1228-31.
5. Lachar, D., et al., *The Brief Psychiatric Rating Scale for Children (BPRS-C): Validity and Reliability of an Anchored Version.* Journal of the American Academy of Child & Adolescent Psychiatry, 2001. 40(3): p. 333-340.
6. Hughes, C.W., et al., *A revised anchored version of the BPRS-C for childhood psychiatric disorders.* J Child Adolesc Psychopharmacol, 2001. 11(1): p. 77-93.
7. Varni, J.W., M. Seid, and C.A. Rode, *The PedsQL™: Measurement Model for the Pediatric Quality of Life Inventory.* Medical Care, 1999. 37(2): p. 126-139.
8. Achenbach, T.M. and L.A. Rescorla, *Manual for the ASBEA school-age forms & profiles*. 2001, Burlington, VT: University of Vermont, Research Center for Children, Youth, & Families.
9. Verhulst, F.C. and J. van der Ende, *Handleiding ASEBA-vragenlijsten voor leeftijden 6-18 jaar*. 2013, Rotterdam: ASEBA Nederland.
10. Bauhuis, O., et al., *MINI KID. De introductie van een Nederlandstalig instrument om DSM-IV-TR diagnoses bij kinderen te stellen.* Kind en adolescent praktijk, 2013. 1: p. 20-26.
11. Sheehan, D.V., et al., *Reliability and validity of the Mini International Neuropsychiatric Interview for Children and Adolescents (MINI-KID).* J Clin Psychiatry, 2010. 71(3): p. 313-26.
12. Ising, H.K., et al., *The Validity of the 16-Item Version of the Prodromal Questionnaire (PQ-16) to Screen for Ultra High Risk of Developing Psychosis in the General Help-Seeking Population.* Schizophrenia Bulletin, 2012. 38(6): p. 1288-1296.
13. Klonsky, E.D. and C.R. Glenn, *Assessing the functions of non-suicidal self-injury: Psychometric properties of the Inventory of Statements About Self-injury (ISAS).* J Psychopathol Behav Assess, 2009. 31(3): p. 215-219.
14. Kerkhof, A.J.F.M., et al., *VOZZ & VOZZ-SCREEN. Vragenlijst over zelfdoding en zelfbeschadiging. Handleiding*. 2015, Amsterdam: Vrije Universiteit Amsterdam.
15. Posner, K., et al., *The Columbia-Suicide Severity Rating Scale: Initial Validity and Internal Consistency Findings From Three Multisite Studies with Adolescents and Adults.* Am J Psychiatry, 2011. 168(12): p. 1266-77.
16. Constantino, J.N. and C.P. Gruber, *Social Responsiveness Scale (SRS)*. 2012, Torrance, CA: Western Psychological Services.
17. Roeyers, H., et al., *SRS-2 Screeningslijst voor autismespectrumstoornissen*. 2015, Amsterdam: Hogrefe Uitgevers.
18. Brown, C. and W. Dunn, *Adolescent-adult sensory profile: user's manual*. 2002, San Antonio: Therapy Skill Builders.
19. Rietman, A., *Adolescent/Adult Sensory Profile-NL, Tieners en Volwassenen 11 t/m 65 jaar, Handleiding*. 2007, Amsterdam: Pearson Clinical.
20. Piquero, A.R., R. MacIntosh, and M. Hickman, *The validity of a self-reported delinquency scale: comparisons across gender, age, race and place of residence.* Sociological methods & research, 2002. 30(4): p. 492-529.
21. Raine, A., et al., *The Reactive-Proactive Aggression Questionnaire: Differential Correlates of Reactive and Proactive Aggression in Adolescent Boys.* Aggress Behav, 2006. 32(2): p. 159-171.
22. Cima, M., et al., *Validation of the Dutch Reactive Proactive Questionnaire (RPQ): Differential Correlates of Reactive and Proactive Aggression From Childhood to Adulthood.* Aggressive Behavior, 2013. 39(2): p. 99-113.
23. Kuntsche, E. and S. Kuntsche, *Development and validation of the Drinking Motive Questionnaire Revised Short Form (DMQ-R SF).* J Clin Child Adolesc Psychol, 2009. 38(6): p. 899-908.
24. Meerkerk, G.J., et al., *The Compulsive Internet Use Scale (CIUS): some psychometric properties.* Cyberpsychol Behav, 2009. 12(1): p. 1-6.
25. Hop, L. and B. Delver, *Jongeren lijden aan Social Media Stress (SMS): Jongeren in de greep van de Sociale Media*. 2012, Amsterdam: Nationale Academie voor Media & Maatschappij.
26. van Rooij, A.J., et al., *Video game addiction test: validity and psychometric characteristics.* Cyberpsychol Behav Soc Netw, 2012. 15(9): p. 507-11.

27. van Baardewijk, Y., et al., *Development and Tests of Short Versions of the Youth Psychopathic Traits Inventory and the Youth Psychopathic Traits Inventory-Child Version.* European Journal of Psychological Assessment, 2010. 26(2): p. 122-128.
28. Ellis, L. and M. Rothbart, *Revision of the Early Adolescent Temperament Questionnaire.* Poster presented at the 2001 Biennal Meeting of the Society for Research in Child Development, 2001.
29. Hartman, C.A., *Nederlandse vertaling van de Early Adolescent Temperament Questionnaire*. 2000: Internal Report, Department of Psychiatry, University Medical Center Groningen, The Netherlands.
30. Bouwmans, C.A.M., S. Schawo, and L. Hakkaart-Van Rooijen, *Handleiding vragenlijst TiC-P kinderen.* 2012, Rotterdam: Institute for Medical Technology Assessment / Erasmus Universiteit Rotterdam.
31. Jellesma, F., M. Meerum Terwogt, and C. Rieffe, *De Nederlandstalige Sense of Coherence vragenlijst voor Kinderen.* gedrag en gezondheid, 2006. 34(1): p. 12-17.
32. Torsheim, T., L.E. Aaroe, and B. Wold, *Sense of coherence and school-related stress as predictors of subjective health complaints in early adolescence: interactive, indirect or direct relationships?* Social Science & Medicine, 2001. 53(5): p. 603-614.
33. Rosenberg, M., *Society and the adolescent self-image*. 1965, Princeton, NJ: Princeton University Press.
34. Kleijn, W.C., G.L.v. Heck, and A.v. Waning, *Ervaringen met een Nederlandse bewerking van de COPE copingvragenlijst.* Gedrag en gezondheid, 2000. 28(4): p. 213-226.
35. Verschuere, B. and H. Tibboel, *De Nederlandstalige versie van de McLean Screening Instrument for borderline personality disorder (MSI-BPD).* Psychologie & Gezondheid, 2011. 39(4): p. 245-250.
36. Zanarini, M.C., et al., *A screening measure for BPD: the McLean Screening Instrument for Borderline Personality Disorder (MSI-BPD).* J Pers Disord, 2003. 17(6): p. 568-73.
37. Anderson, J.L., M. Sellbom, and R.T. Salekin, *Utility of the Personality Inventory for DSM-5-Brief Form (PID-5-BF) in the Measurement of Maladaptive Personality and Psychopathology.* Assessment, 2018. 25(5): p. 596-607.
38. De Clercq, B., et al., *The hierarchical structure and construct validity of the PID-5 trait measure in adolescence.* J Pers, 2014. 82(2): p. 158-69.
39. Boterhoven de Haan, K.L., et al., *Reliability and validity of a short version of the general functioning subscale of the McMaster Family Assessment Device.* Fam Process, 2015. 54(1): p. 116-23.
40. Epstein, N.B., L.M. Baldwin, and D.S. Bishop, *THE McMASTER FAMILY ASSESSMENT DEVICE*.* Journal of Marital and Family Therapy, 1983. 9(2): p. 171-180.
41. Vermulst, A., et al., *Handleiding OBVL*. 2015, Delft: Eburon Uitgeverij B.V.
42. Straus, M.A. and M.J. Mattingly, *A short form and severity level types for the parent-child conflict tactics scales*. 2007: Family Research Laboratory, University of New Hampshire, Durham.
43. Lange, A., *De Ouder-Kind Interactievragenlijst - Revised. OKIV-R, verantwoording en handleiding* 2001, Houten: Bohn Stafleu van Loghum.
44. Veerman, J.W., et al., *Vragenlijst Gezinsfunctioneren volgens Ouders (VGFO). Handleiding*. 2016, Nijmegen: Praktikon.
45. Loeber, R., et al., *Antisocial behavior and mental health problems: Explanatory factors in childhood and adolescence*. 1998, Mahwah, NJ: Lawrence Erlbaum.
46. Zimet, G.D., et al., *The Multidimensional Scale of Perceived Social Support.* Journal of Personality Assessment, 1988. 52: p. 30-41.
47. Bernstein, D.P., et al., *Development and validation of a brief screening version of the Childhood Trauma Questionnaire.* Child Abuse Negl, 2003. 27(2): p. 169-90.
48. Thombs, B.D., et al., *A validation study of the Dutch Childhood Trauma Questionnaire-Short Form: Factor structure, reliability, and known-groups validity.* Child Abuse & Neglect, 2009. 33(8): p. 518-523.
49. Tellegen, P.J. and J.A. Laros, *Snijder-Oomen Niet-verbale intelligentietest SON-R 6-40. I. Verantwoording. II. Instructies. III. Nederlands-Duitse normen 2010.* 2011, Amsterdam: Hogreve uitgevers.
50. Bechara, A., et al., *Insensitivity to future consequences following damage to human prefrontal cortex.* Cognition, 1994. 50(1): p. 7-15.
51. Buelow, M.T. and J.A. Suhr, *Construct Validity of the Iowa Gambling Task.* Neuropsychology Review, 2009. 19(1): p. 102-114.
52. Huizinga, M. and D. Smidts, *BRIEF Screener Vragenlijst voor snelle screening van executieve functies bij kinderen en jongeren. Nederlandse bewerking*. 2016, Amsterdam: Hogrefe Uitgevers.
53. van Hemert, A.M., *Lichamelijke Klachten Vragenlijst*. 2003, Leiden: Leids Universitair Medisch Centrum.
54. Magaña, A.B., et al., *A brief method for assessing expressed emotion in relatives of psychiatric patients.* Psychiatry Research, 1986. 17(3): p. 203-212.
55. Meerwaldt, R., et al., *Simple noninvasive measurement of skin autofluorescence.* Ann N Y Acad Sci, 2005. 1043: p. 290-8.

56. van den Bosch, G.E., et al., *Thermal quantitative sensory testing in healthy Dutch children and adolescents standardized test paradigm and Dutch reference values*. BMC Pediatr, 2017. 17(1): p. 77.
57. Endicott, J., et al., *The Global Assessment Scale: A Procedure for Measuring Overall Severity of Psychiatric Disturbance*. Archives of General Psychiatry, 1976. 33(6): p. 766-771.
58. Sheehan, D.V., et al., *The Mini-International Neuropsychiatric Interview (M.I.N.I.): the development and validation of a structured diagnostic psychiatric interview for DSM-IV and ICD-10*. J Clin Psychiatry, 1998. 59 Suppl 20: p. 22-33;quiz 34-57.
59. van Vliet, I. and E. de Beurs, *Het Mini Internationaal Neuropsychiatrisch Interview (MINI): Een kort gestructureerd diagnostisch psychiatrisch interview voor DSM-IV- en ICD-10 stoornissen*. Tijdschrift voor psychiatrie, 2007. 49(6): p. 393-397.
60. de Beurs, E., *Brief Symptom Inventory, handleiding*. 2004, Leiden: Pits Publishers.
61. Moran, P., et al., *Standardised Assessment of Personality – Abbreviated Scale (SAPAS): Preliminary validation of a brief screen for personality disorder*. The British Journal of Psychiatry, 2003. 183(3): p. 228-232.
62. Hesse, M. and P. Moran, *Screening for personality disorder with the Standardised Assessment of Personality: Abbreviated Scale (SAPAS): further evidence of concurrent validity*. BMC Psychiatry, 2010. 10(1): p. 10.
63. Bouwmans, C.A.M., L.H. Roijen, and A. Institute for Medical Technology, *TiC-P volwassenen: vragenlijst over zorggebruik en productiviteitsverliezen bij psychische aandoeningen : handleiding (update 2012)*. 2013: Institute for Medical Technology Assessment, Erasmus Universiteit Rotterdam.
